# Gene’s expression underpinning the divergent predictive value of [18F]F-fluorodeoxyglucose and prostate-specific membrane antigen positron emission tomography in primary prostate cancer: a bioinformatic and experimental study

**DOI:** 10.1186/s12967-022-03846-1

**Published:** 2023-01-04

**Authors:** Matteo Bauckneht, Cecilia Marini, Vanessa Cossu, Cristina Campi, Mattia Riondato, Silvia Bruno, Anna Maria Orengo, Francesca Vitale, Sonia Carta, Silvia Chiola, Sabrina Chiesa, Alberto Miceli, Francesca D’Amico, Giuseppe Fornarini, Carlo Terrone, Michele Piana, Silvia Morbelli, Alessio Signori, Paola Barboro, Gianmario Sambuceti

**Affiliations:** 1 Department of Health Sciences, University of Genoa, 16132 Genoa, Italy; 2 Nuclear Medicine Unit, IRCCS, Ospedale Policlinico San Martino, 16132 Genoa, Italy; 3 CNR, Institute of Molecular Bioimaging and Physiology (IBFM), 20054 Milan, Italy; 4 LISCOMP Lab, Department of Mathematics (DIMA), University of Genoa, 16132 Genoa, Italy; 5 Department of Experimental Medicine, Human Anatomy, University of Genoa, 16132 Genoa, Italy; 6 CNR-SPIN Genoa, 16132 Genoa, Italy; 7 Medical Oncology Unit 1, IRCCS Ospedale Policlinico San Martino, 16132 Genoa, Italy; 8 Department of Urology, IRCCS Ospedale Policlinico San Martino, 16132 Genoa, Italy; 9 Department of Surgical and Diagnostic Integrated Sciences (DISC), University of Genova, 16132 Genoa, Italy; 10 Proteomic and Mass Spectrometry Unit, IRCCS, Ospedale Policlinico San Martino, 16132 Genoa, Italy

**Keywords:** Prostate cancer, Glucose metabolism, Prostate-specific membrane antigen, Positron emission tomography, Prognosis

## Abstract

**Background:**

Positron Emission Tomography (PET) imaging with Prostate-Specific Membrane Antigen (PSMA) and Fluorodeoxyglucose (FDG) represent promising biomarkers for risk-stratification of Prostate Cancer (PCa). We verified whether the expression of genes encoding for PSMA and enzymes regulating FDG cellular uptake are independent and additive prognosticators in PCa.

**Methods:**

mRNA expression of genes involved in glucose metabolism and PSMA regulation obtained from primary PCa specimens were retrieved from open-source databases and analyzed using an integrative bioinformatics approach. Machine Learning (ML) techniques were used to create predictive Progression-Free Survival (PFS) models. Cellular models of primary PCa with different aggressiveness were used to compare [18F]F-PSMA-1007 and [18F]F-FDG uptake kinetics in vitro. Confocal microscopy, immunofluorescence staining, and quantification analyses were performed to assess the intracellular and cellular membrane PSMA expression.

**Results:**

ML analyses identified a predictive functional network involving four glucose metabolism-related genes: ALDOB, CTH, PARP2, and SLC2A4. By contrast, FOLH1 expression (encoding for PSMA) did not provide any additive predictive value to the model. At a cellular level, the increase in proliferation rate and migratory potential by primary PCa cells was associated with enhanced FDG uptake and decreased PSMA retention (paralleled by the preferential intracellular localization).

**Conclusions:**

The overexpression of a functional network involving four glucose metabolism-related genes identifies a higher risk of disease progression since the earliest phases of PCa, in agreement with the acknowledged prognostic value of FDG PET imaging. By contrast, the prognostic value of PSMA PET imaging is independent of the expression of its encoding gene FOLH1. Instead, it is influenced by the protein docking to the cell membrane, regulating its accessibility to tracer binding.

**Supplementary Information:**

The online version contains supplementary material available at 10.1186/s12967-022-03846-1.

## Introduction

Prostate cancer (PCa) manifests a broad spectrum of intrinsic biological aggressiveness, paralleled by significant inter-patient heterogeneity [1]. While 5-year survival rates are excellent for localized PCa, lifespan is limited for patients with distant tumour burden [2]. Moreover, local relapse and distant metastases occur during the clinical follow-up in 20–30% of PCa patients initially treated with curative intent [3–5]. This variable clinical behaviour asks for the development of biomarkers potentially able to improve risk stratification, mostly in newly diagnosed treatment-naïve patients.

In the last years, PCa initial staging has been profoundly reshaped by the introduction of [18F]F- or [68 Ga]Ga-labelled radiotracers targeting the Prostate-Specific Membrane Antigen (PSMA) [6, 7], a type II integral membrane glycoprotein encoded by the FOLH1 gene whose expression is markedly higher in PCa than in normal prostatic tissues [8]. Mapping the PSMA distribution by Positron Emission Tomography/Computed Tomography (PET/CT) imaging couples the improved capability to define the presence and localization of cancer cells with the estimation of antigen expression and tumour volume. Several studies have already reported a direct correlation between the degree of PSMA tracer uptake and histopathological features of disease severity, including the Gleason Score [9–12]. Coherently, the higher the PSMA tracer uptake by the primary tumour, the lower the long-term clinical outcome [13]. However, the PSMA PET prognostic penetrance is challenged by the acknowledged limitation of PET imaging that underestimates tracer concentration in small tumours and by the notion that 5–10% of clinically relevant PCa do not express this protein [14, 15].

[18F]F-Fluorodeoxyglucose (FDG) is one of the most used PET tracers in oncology. Mapping the high glycolytic rate (also termed the Warburg effect), it displays most solid cancers' proliferative and migratory potential [16]. Differently from early studies [17, 18], recent data reported a high prognostic power of FDG PET in PCa, at least in the metastatic castration-resistant phase [18–23], which is characterized by a higher prevalence of glucose-avid less differentiated neoplastic cells insensitive to androgen deprivation [24]. Prognostic insights provided by PSMA and FDG PET imaging may thus be complementary, reflecting the presence of different cancer phenotypes in different phases of the disease.

The present study tested this hypothesis through a multidisciplinary approach. Using a bioinformatics approach, we verified whether the expression of genes encoding for PSMA and enzymes regulating glucose metabolism are independent and additive outcome predictors in patients with newly diagnosed PCa. Thereafter, we compared PSMA and FDG uptake kinetics in validated cellular models of primary PCa with different aggressiveness grades.

## Materials and methods

### Genes selection

The glucose metabolism-related gene set was downloaded from the Kyoto Encyclopedia of Genes and Genomes (KEGG), a meta-database used to integrate information with genomes, diseases, and biological pathways [25]. FOLH1 and its interactors in PCa were retrieved from the Protein Interaction Network Analysis (PINA) platform (https://omics.bjcancer.org/pina) using The Cancer Genome Atlas Prostate Adenocarcinoma (TCGA-PRAD) dataset. This inquiry provided us with 122 genes that are reported in Additional file 1: Table S1.

### Construction and statistical analyses of predictive models through machine learning techniques

Analyzing the cBioPortal database (https://www.cbioportal.org/), we searched studies reporting data associated with the mRNA expression for all 122 investigated genes in primary PCa specimens obtained after surgery in primary PCa patients. These data were available for 493 PCa patients in the TCGA-PRAD [26] dataset. The study focused on z-score mRNA expression relative to diploid samples (RNA Seq V2 RSEM) compared with the follow-up data of all PCa patients (details about the expression profile are reported in the Additional file 2). Progression-Free Survival (PFS) was used as the primary clinical endpoint as the most reliable outcome for PCa [26] and was defined as the interval between the date of diagnosis and the date of the new event returned, including the progression of the cancer, local recurrence, distant metastases, or death from the cancer. All data were retrieved from open resources, and thus no ethical issues were involved. Predictive models of PFS were set up using two Machine Learning (ML) techniques: Random Forest [27] and a hybrid version of Lasso [28], where an unsupervised fuzzy C-means step chooses the threshold for the definition of the event. Both techniques provide a weight representing its contribution to the predictive model for each gene expression to retain the more significant predictors and discard the ones with negligible weights. To test the robustness of obtained ranking, we applied a bootstrap analysis. Data encompassed 493 subjects, 93 of whom (18.9%) displayed disease progression during the follow-up. This cohort was divided into a training set containing 329 patients (67%) and a test set containing the remaining 164 (33%). This division was performed randomly, with the only constraint of respecting the event rates in the training and test sets. The random split was repeated 100 times. We then trained the ML algorithms on the training sets and tested their predictive performance on the test sets. For all 100 realizations the two ML methods allow an automatic ranking of the input features and the identification of the features that mostly impact the prediction. These features were selected for a multivariable Cox regression model. No further clinic-pathological data were added to the multivariable analysis to obtain a purely genetic model. Only those with a *p-value* < 0.05 in the multivariable model were retained in the final model. To stabilize the coefficient of each feature included in the multivariable model, a bootstrap approach with 500 replications was applied. The concordance of Harrell's c-index was also reported to evaluate the discriminative ability of the multivariable model. To consider the possible overfitting during the building and estimation of the prognostic model, a bias-corrected estimate (optimism correction) of the c-index was reported. The weight of each gene resulting in statistically significant was extracted from the multivariate logistic regression model. Obtained results were used to create a formula, resulting in a genetic score able to predict PFS. Clinical characteristics of PCa patients divided according to the genetic score result were compared using a t-test for continuous variables or a Chi-square test to compare proportions. Moreover, the obtained genetic score was included in a univariate and multivariate logistic regression model built to predict PFS including the available clinical characteristics retrieved from the TCGA-PRAD dataset. In particular, age at diagnosis, race, histopathological tumour type, local (T)- and nodal (N)-status at histopathology. The obtained genetic score's predictive power was then validated using an external dataset retrieved from the cBioPortal database (PRAD-MSKCC). MedCalc 19.4 (MedCalc Software, Ostend, Belgium), Stata v.16 (StataCorp. 2019) and R (v.4.0.2; Rcore Team) were used for the computation.

### Tissue-specific functional networks

To explore the potential effects of the involved genes, we planned to identify their interactors (Additional file 1: Table S2), building a tissue-specific functional network using data from the PINA platform (https://omics.bjcancer.org/pina/). The protein–protein interaction network was created by extracting data from five manually curated databases (IntAct, MINT, BioGRID, DIP, HPRD) and unified using database integration techniques [29]. To build the cancer-specific network we used the “cancer-context” utility with the following query parameters: the TCGA PRAD transcriptomic profiles, tumour type specificity score > 2 and Spearman correlation coefficient > 0.1. In the obtained figures, the edge width is proportional to the correlation coefficient. This network’s first ten central genes (hubs) were determined with PINA network analysis utility by eigenvector centrality measure. Enrichment analysis and visualization of Gene Ontology (GO) terms in biological process and molecular function categories were performed using ClueGO Cytoscape plugin (significance: *p-value* < 0.05). A network diagram was created by grouping GO terms using the kappa score (> 0.3).

### Human PCa LNCaP cellular model

LNCaP cells, obtained from the American Type Culture Collection (CRL-1740), were maintained in RPMI 1640 medium supplemented with 10% FBS, 1% glutamine, 10 mM HEPES, 1 mM sodium pyruvate, 4.5 mg/ml glucose, 1% penicillin and 1% streptomycin, in poly-D-lysine coated flask. Different degrees of PCa severity were reproduced using a previously validated model, implying the evaluation of LNCaP with less than 33 passages (LNCaP-30) and with over 80 passages (LNCaP-80) as models of low and high aggressiveness, respectively [30, 31]. According to local legislation, no ethical approval was needed for in vitro experiments.

### In vitro* kinetics of [18F]F-FDG and [18F]F-PSMA-1007 uptake*

Uptake kinetics of [18F]F-FDG and [18F]F-PSMA-1007 were estimated in both LNCaP-30 and LNCaP-80 cell cultures. A total of six experiments were performed for each culture type. Both tracers were synthesized by the radiopharmacy lab of the Nuclear Medicine facility of IRCCS Ospedale Policlinico San Martino and passed the due quality controls. Tracer uptake of each cell culture was evaluated using the LigandTracer White® instrument (Ridgeview, Uppsala, SE) according to our validated procedure [32–35]. The device consists of a beta-emission detector and a rotating platform harbouring a standard Petri dish. The rotation axis is inclined at 30° from the vertical so that the organ alternates its position from the nadir (for incubation) to the zenith (for counting) every minute for an experiment duration of 120 min. One hundred seventy-five thousand cells were seeded the day before the experiments and cultured under standard conditions. Soon before each experiment, the culture medium was replaced with 3 mL DMEM containing glucose at 5.5 mM and either [18F]F-PSMA-1007 (1.8–2.2 MBq/ml) or [18F]F-FDG (1.8–2.2 MBq/ml). Each experiment was preceded by a calibration procedure documenting that recovered counts were 3 ± 0.07% of the source emission in all cases. Accordingly, culture radioactivity content at each time was normalized as a fraction of the administered dose. [18F]F-FDG accumulation was analyzed considering the standard Sokoloff assumption of an irreversible pool for tracer accumulation. The model was thus tested using the conventional graphical approach described by Patlak et al. [36], identifying the straight line described by the following equation:

$$\frac{{FDG}_{cells}}{{FDG}_{DMEM}}= a \frac{{\int }_{0}^{t}{FDG}_{DMEM(t)}}{{FDG}_{DMEM(t)}}+b$$

where $\frac{{FDG}_{cells}}{{FDG}_{DMEM}}$ represents the fraction of administered dose taken up by PCa cells, while FDG_DMEM_ is the tracer concentration in the medium and thus the administered dose subtracted by FDG cells at each time t. By contrast, $a$ (the slope of the regression line) represents the tracer accumulation rate multiplied by the glucose concentration to estimate the glucose consumption of the cell culture.

Kinetic analysis of [18F]F-PSMA-1007 uptake was performed according to the conventional one-phase association ligand-receptor kinetics. For this purpose, we used the non-linear regression analysis routine of the Prism GraphPad software package fitting each experimental curve according to the following function:

$${A}_{t}={A}_{max} \times (1-{e}^{kt})$$

where ${A}_{t}$ is the observed fraction of administered dose in cell culture at each time *t*, ${A}_{max}$ is its maximal asymptotic plateau phase, representing the number of maximal ligand sites, and *k* represents the receptor-ligand affinity constant.

### PSMA expression pattern by confocal microscopy

**Fig. 1:**
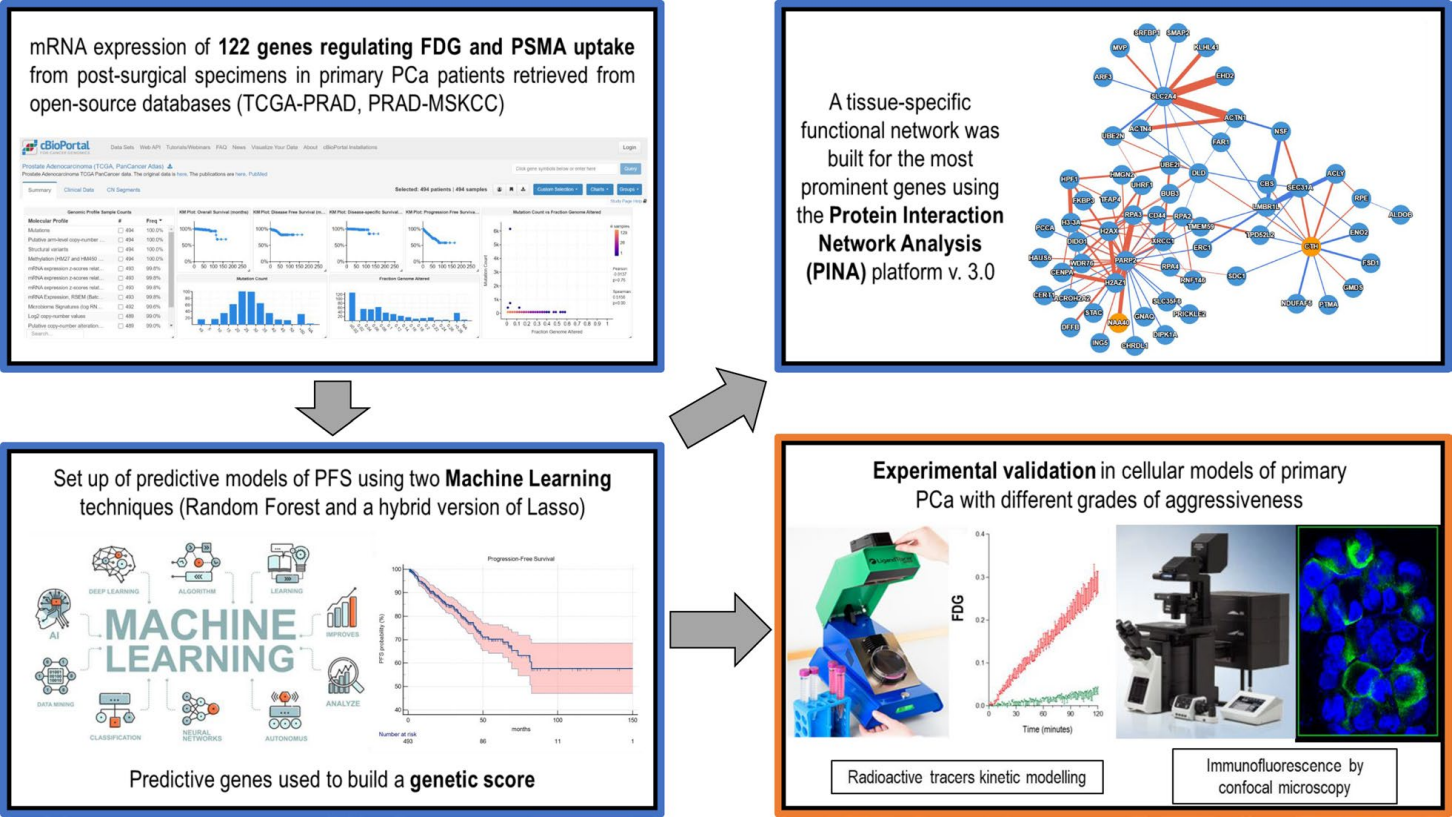
Graphical summary of the study experiments. The four panels of the figure summarise the bioinformatics (blue frame) and experimental (orange frame) study experiments. The grey arrows display the timeline of the study

Intracellular and plasma membrane PSMA expression was assessed on LNCaP-30 and LNCaP-80 cells cultured on glass coverslips by immunofluorescence, using an anti-PSMA monoclonal antibody (GCP-05) followed by Goat anti-Mouse Alexa 488 antibody (both from ThermoFisher). The abundance of plasma membrane docked PSMA was evaluated in fresh and un-permeabilized cells. For PSMA intracellular content, immunofluorescence cells were fixed with 3.7% paraformaldehyde and permeabilized with 0.1% Triton X-100. Cells were counterstained with DAPI, and the slides were mounted with Mowiol mounting medium for analysis on the SP2-AOBS confocal microscope (Leica Microsystems, Mannheim, Germany). Each experiment was performed in triplicate, and immunofluorescence computation was performed using appropriate software packages (ImageJ, NIH and Leica software). A graphical summary of the study experiments and their timeline is represented in Fig. 1.

## Results

### Gene’s expression predictive model

**Fig. 2:**
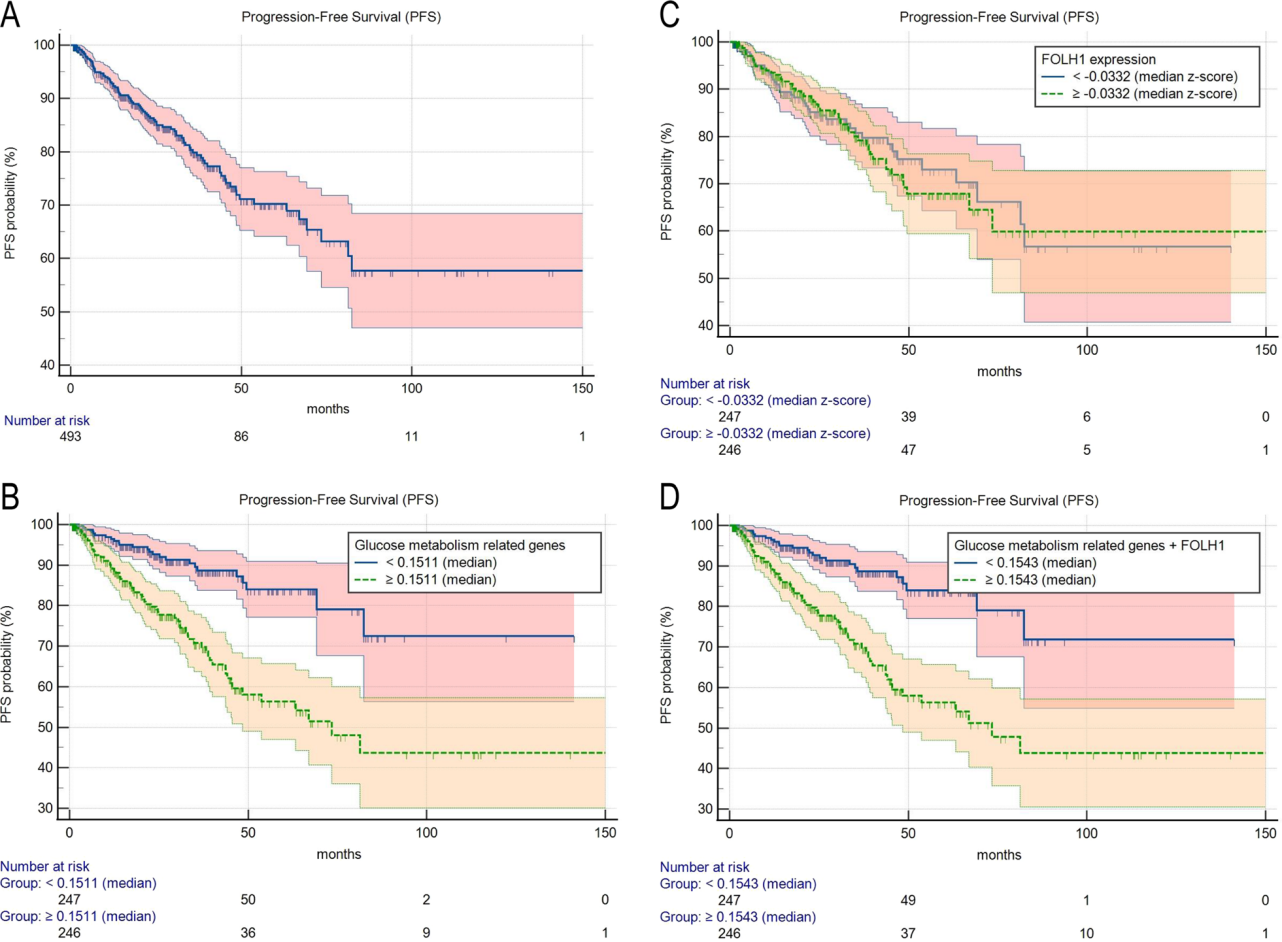
The predictive power of genetic scores in PCa. **A** displays the PFS function of the entire study cohort. Subsequent panels display Kaplan–Meier curves for PFS according to the genetic score built using the four most prominent glucose metabolism-related genes (PARP2, SLC2A4, CTH, ALDOB) (**B**), according to FOLH1 expression (**C**), and FOLH1 forcedly added to the genetic score described above (**D**), respectively. Median values were set as cut-off values to binarize data

Glucose metabolism-related and PSMA expression-related genes in PCa (Additional file 1: Table S1) were analyzed using integrative bioinformatics analysis. For this purpose, we created the top 10 ranking hubs for Random Forest and Hybrid Lasso, averaging and ordering the 100 rankings provided by the bootstrap analysis. Then, we considered the two top 10 rankings and looked for the features present in both. The most prominent genes were: ALDOB, CTH, PARP2, and SLC2A4. The weight of each gene was extracted from the multivariate logistic regression model built to predict PFS (Table 1, see also Fig. 2A for the PFS function of the entire study cohort). Obtained results were used to create a genetic score according to the following formula:

$$ 0.254{\text{*PARP}}2{ }{-}{ }0.733{\text{*SLC}}2{\text{A}}4{ }{-}{ }0.372{\text{*CTH }} + { }0.140{\text{*ALDOB}} $$

**Table 1:** Multivariate logistic regression model built to predict PFS

	Original set (n = 493)		Bootstrap (500 replication)		
Gene	β-coefficient ± SE	p-value	β-coefficient ± SE	HR (95% CI)	p-value
PARP2	0.247 ± 0.0788	0.002	0.254 ± 0.0914	1.29 (1.08–1.54)	0.005
SLC2A4	− 0.713 ± 0.234	0.002	− 0.733 ± 0.244	0.48 (0.30–0.78)	0.004
CTH	− 0.370 ± 0.118	0.002	− 0.372 ± 0.144	0.69 (0.52–0.91)	0.009
ALDOB	0.144 ± 0.0564	0.011	0.140 ± 0.0588	1.15 (1.03–1.29)	0.024
FOLH1	− 0.0888 ± 0.112	0.43	− 0.0943 ± 0.110	0.91 (0.73–1.13)	0.44
Harrell’s C-index	0.714		0.701^a^		

^a^Corrected for Optimism

Once divided according to the median value, the resulting score significantly predicted PFS in PCa patients (mPFS not reached vs 73.4 months, p < 0.0001, Fig. 2B). PCa patients belonging to the low- and high-risk groups according to the glucose-metabolism-related genetic score did not differ in age at diagnosis (60.61 ± 7.3 vs. 61.45 ± 6.2, respectively, p = 0.173) and race (95.5% vs. 95.5% white race, respectively, p = 1.00). By contrast, the two subgroups diverged according to the histopathological tumour type (0.4% vs. 2.6% non-acinar subtype, respectively, p = 0.003), T-status (24.5% vs. 37.2% ≥ T3a, respectively, p < 0.0001) and N-status (4.8% vs. 13.8% N1, respectively, p < 0.0001) at histopathology. Once included in a univariate and multivariate Cox regression model together with the available clinical characteristics of the TCGA-PRAD dataset (age at diagnosis, race, histopathological tumor type, T- and N-status at histopathology), the glucose-metabolism-related genetic score significantly and independently predicted PFS together with the T-status at histopathology (Table 2). The same glucose-metabolism-related genetic score significantly predicted PFS also in the validation dataset (PRAD-MSKCC, HR = 1.75, p = 0.011).

**Table 2:** Univariate and multivariate Cox regression model built to predict PFS including the genetic score and clinical characteristics of PCa patients

Variable		Univariate		Multivariate	
		HR (95% CI)	p-value	HR (95% CI)	p-value
Glucose-metabolism-related genetic score	< 0.1511	1.00 (ref)	–	1.00 (ref)	–
	≥ 0.1511	3.42 (2.15–5.45)	** < 0.0001**	2.81 (1.69–4.62)	** < 0.0001**
Age at diagnosis (years)	< 61	1.00 (ref)	–		
	≥ 61	1.62 (0.77–1.74)	0.469		
Race	White	1.00 (ref)	–		
	Black or African American	0.001 (0.001–255.10)	0.957		
Histopathological tumour type	Acinar	1.00 (ref)	–		
	Other type	2.04 (0.74–5.57)	0.162		
T-status	< T2c	1.00 (ref)	–	1.00 (ref)	–
	≥ T3a	3.69 (2.09–6.54)	** < 0.0001**	3.06 (1.61–5.85)	**0.0007**
N-status	0	1.00 (ref)	–		
	1	1.81 (1.11–2.96)	**0.016**		

Statistically significant p values are highlighted in bold

Unlike glucose metabolism-related genes, FOLH1 expression did not significantly predict PFS (p = 0.81, Fig. 2C). FOLH1 was then forcedly added to the genetic score described above, according to the following formula:

$$ 0.254{\text{*PARP}}2{ }{-}{ }0.733{\text{*SLC}}2{\text{A}}4{ }{-}{ }0.372{\text{*CTH }} + { }0.140{\text{*ALDOB }}{-}{ }0.0943{\text{*FOLH}}1 $$

As expected, FOLH1 addition did not substantially modify the predictive value of the genetic score (mPFS not reached vs 73.4 months, p < 0.0001, Fig. 2D).

### Tissue-specific functional networks

**Fig. 3:**
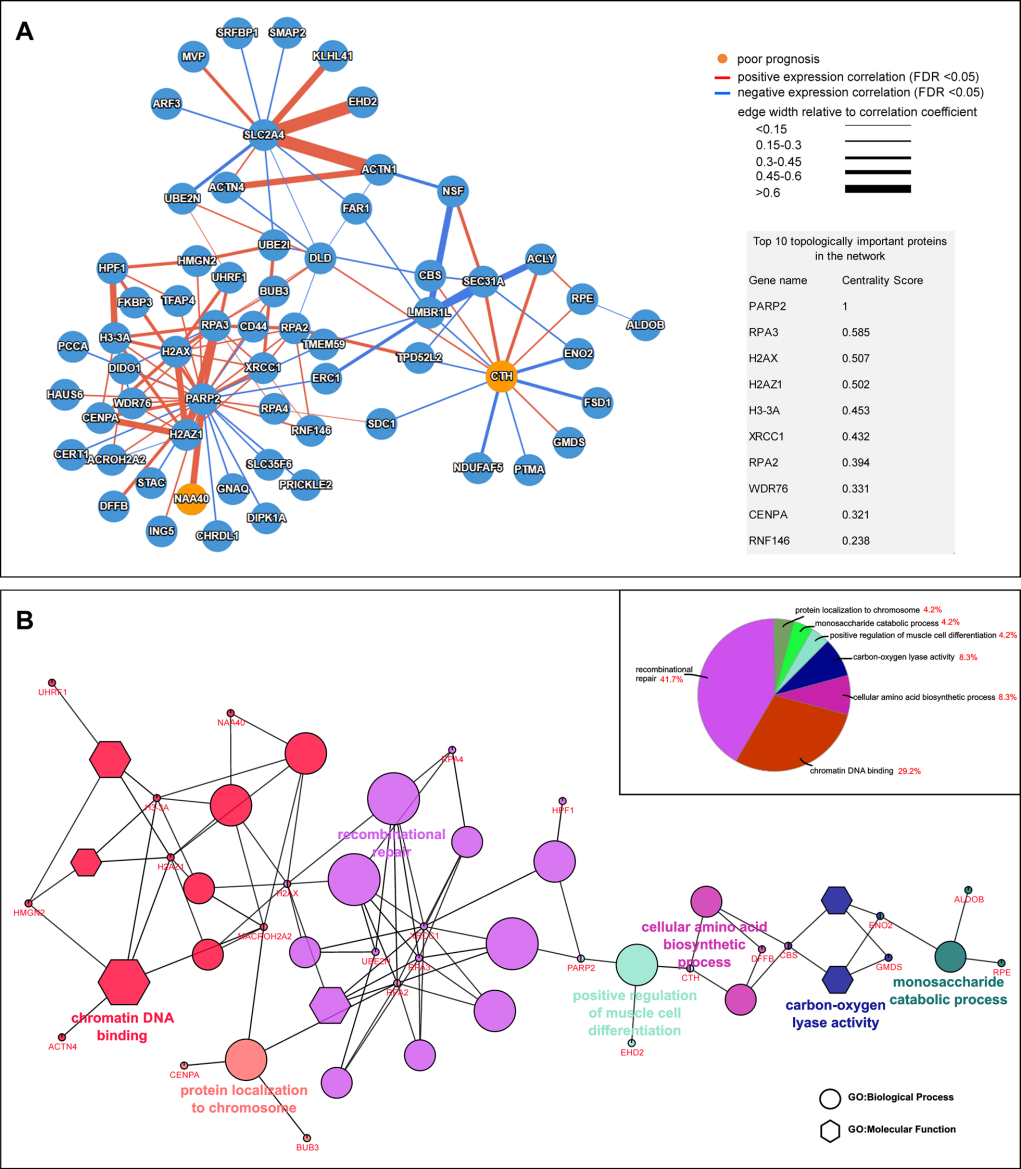
Functional network of PARP2, SLC2A4, CTH and ALDOB genes in PCa. **A** functional network of the four genes and their interactors reported in Additional file 1: Table S2. The edge width is proportional to the correlation coefficient. The top 10 central genes and their centrality score are listed in the inset table. **B** graphical overview of ClueGO results of genes reported in Additional file 1: Table S2. The pie diagram in the inset shows the percentage of the GO terms associated with the groups

The subsequent analysis was performed to evaluate the functional relationships of ALDOB, CTH, PARP2, and SLC2A4 and their interactors in PCa (Additional file 1: Table S2). The resulting protein–protein interaction network retrieved from the TCGA-PRAD dataset by PINA, showed a good closeness between them (clustering coefficient 0.36) (Fig. 3A). The topological distribution of each protein (node) was estimated to infer its centrality in the network. Among the first ten central nodes of the network (inset table in Fig. 3A), PARP2 had the highest centrality score. Performing the functional enrichment analysis using ClueGo, we explored the biological and functional role of ALDOB, CTH, PARP2, and SLC2A4 in PCa (inset pie of Fig. 3B). Although PARP2, SLC2A4 and ALDO8 are genes involved in glucose metabolism and CTH is related to the cysteine biosynthetic process, recombinational repair and chromatin DNA binding were the most relevant GO terms associated with the four genes interactors. Interestingly, performing the same analyses using PARP2, SLC2A4, CTH, ALDO8 and FOLH1, any significant difference was found in the functional network (Additional file 3: Figure S1A-B, Additional file 2: Figure S1 legend), while, as expected, the ClueGO enrichment showed an increase in the cellular amino acid biosynthetic process (from 8.3% to 13.2%, inset pie of Additional file 3: Figure S1B, Additional file 2: Figure S1 legend).

### Opposite kinetics of [18F]F-FDG and [18F]F-PSMA-1007 in LNCaP-30 and LNCaP-80 cell cultures

**Fig. 4:**
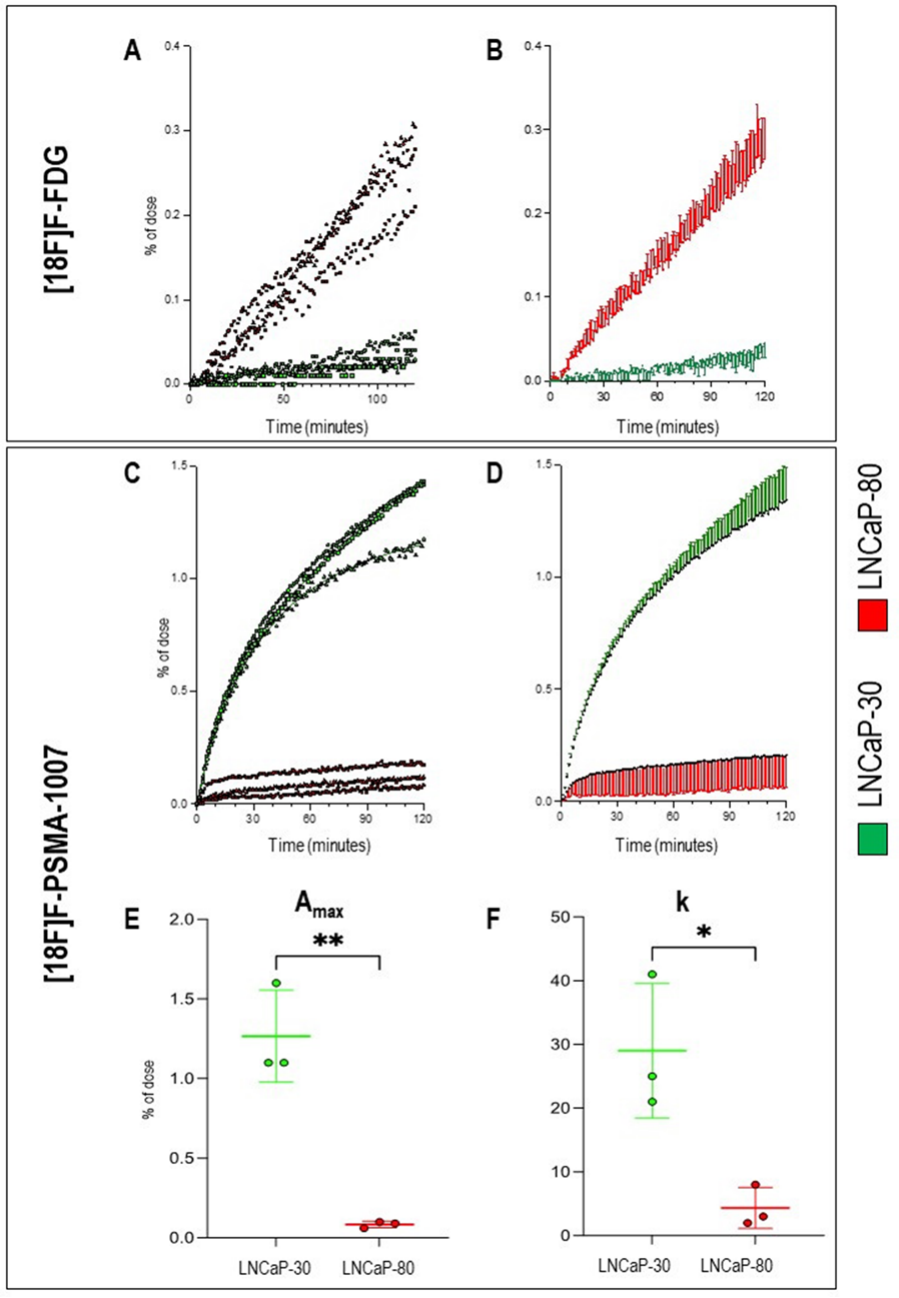
Opposite kinetics of [18F]F-FDG and [18F]F-PSMA-1007 in LNCaP-30 and LNCaP-80 cell cultures. **A**–**B** display row time activity curves, and mean + SD curves in LANCaP-30 (green) and LANCap-80 (red) cells exposed to [18F]F-FDG for 120 min under the LigandTracer instrument, respectively. **C**–**D** display the same curves in LANCaP-30 (green) and LANCap-80 (red) cells exposed to [18F]F-PSMA-1007. **E**–**F** show the difference between the two experimental models in terms of number of accessible receptors and receptor-ligand affinity constant, respectively. Data are shown as the mean ± SD. n = 3 experiments per group, with each value defined in triplicate. * = *p-value* < 0.05; ** = *p-value* < 0.01

We thus investigated the FDG and PSMA kinetics in LNCaP-30 and LNCaP-80 cultures as cellular models of primary PCa with different grades of biological aggressiveness [30, 31]. [18F]F-FDG uptake was markedly slower in LNCaP-30 than in LNCaP-80 cultures. This difference was observed in the analysis of raw time-activity curves and Patlak regression lines, which documented an almost halved rate of tracer accumulation in the former compared to the latter PCa cellular models (1.4 ± 0.2 × 10–7 min^−1^ vs 2.7 ± 0.2 min^−1^, Fig. 4A, B). This finding suggested that the already documented increase in proliferation rate and migratory potential of LNCaP cells exposed to a high number of passages [30, 31] is paralleled by increased avidity for the glucose analogue. By contrast, [18F]F-PSMA-1007 kinetics showed the opposite behaviour, being faster in LNCaP-30 than in LNCaP-80 cultures (Fig. 4C, D). In all experiments, the kinetics of ^18^F-PSMA-1007 accumulation well agreed with the ligand-receptor interaction model showing a high adherence of the fitted curve with the experimental data (R squared values > 0.95 in all cases). This analysis showed a 15-fold decrease in the number of accessible receptors (Fig. 4E) and an almost fivefold decrease in the affinity constant k (Fig. 4F) in LANCap-80 compared to LANCap-30 cells. Therefore, LNCaP cells exposed to a high number of passages showed a reduced affinity for the PSMA-targeted radioactive probe.

### PSMA expression patterns in LNCaP-30p and LNCaP-80 cells

**Fig. 5:**
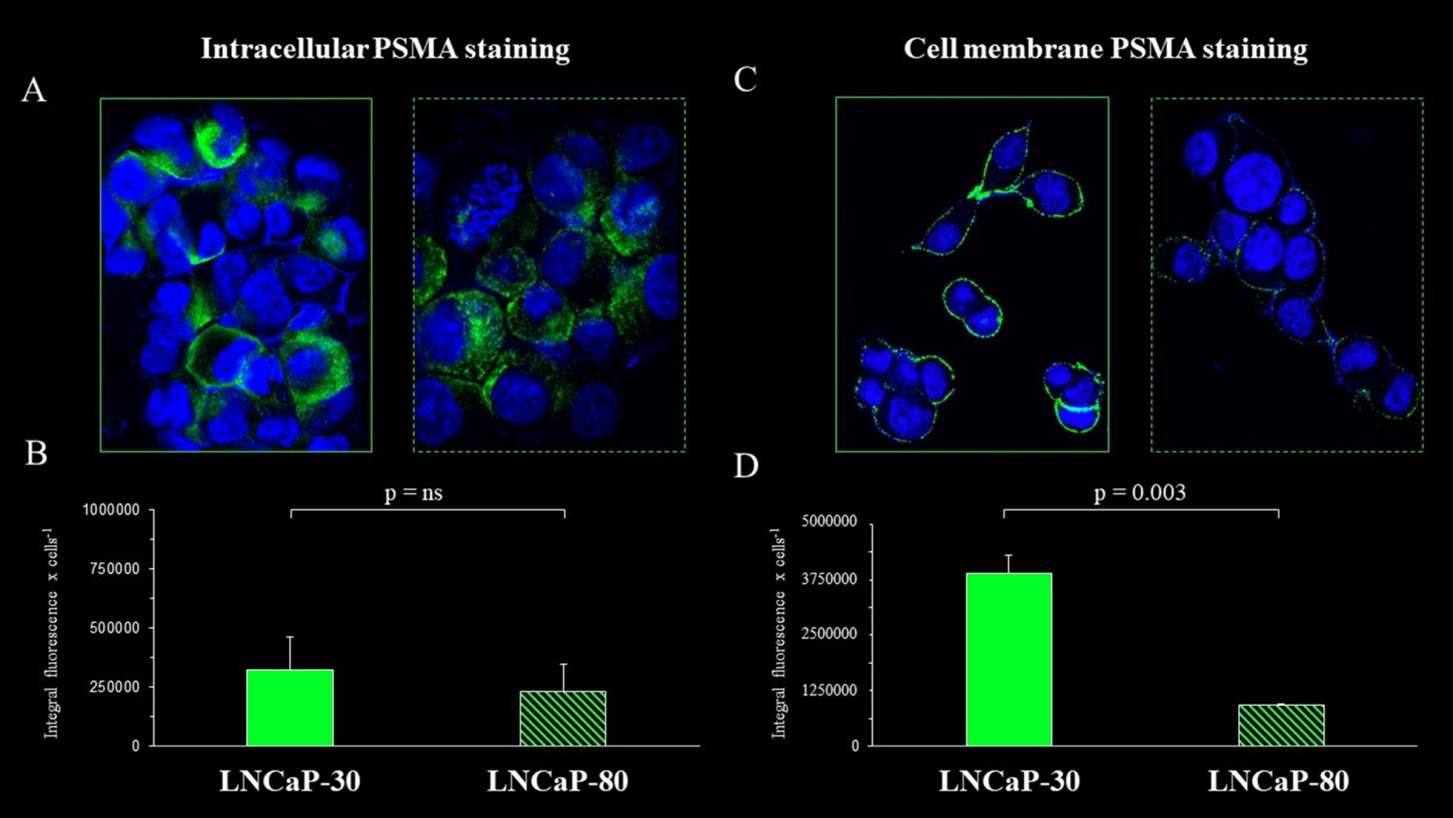
Immunofluorescence staining and quantification analysis of PSMA expression patterns in LANCaP cells with different grades of aggressiveness. Representative confocal microscopy images of LNCaP-30 (solid line) and LNCaP-80 (dashed line) stained with anti-PSMA (green fluorescence) and DAPI (nuclei, blue fluorescence) in permeabilized (**A**) or intact (**C**) cells. Quantification of PSMA fluorescence in permeabilized (**B**) or intact (**D**) cells. Fluorescence was quantified by using ImageJ analysis software. Values of integral fluorescence cell are evaluated in fields containing at least 5 cells each, and are expressed as means ± SD. n = 3 experiments per group. *ns* not significant

The low [18F]F-PSMA-1007 uptake in the LNCaP-80 cellular model apparently disagreed with the negligible prognostic penetrance of FOLH1 expression in the bioinformatics analysis. We thus used an immunofluorescence approach to intact or permeabilized cells to identify the potential occurrence of different PSMA localizations in LNCaP-30 and LNCaP-80 cells. Fluorescence intensity evaluated in permeabilized cells (resulting from both cytosolic and membranous PSMA content) was superimposable in the two cell lines (Fig. 5A, B), suggesting a similar FOLH1 genetic expression. However, when intact cells were evaluated, the fluorescence signal at the cell surface was markedly higher in LNCaP-30 than in LNCaP-80 cells (Fig. 5C, D). Therefore, the two cell lines' different aggressiveness was associated with a post-transcriptional shift modifying the plasma membrane docking of the protein.

## Discussion

Our bioinformatic analysis of a public database documented a prognostic power of four genes (PARP2, SLC2A4, CTH, ALDOB) dedicated to glucose metabolism in PCa. Instead, this capability did not characterize FOLH1 expression. Similarly, the increase in proliferation rate and migratory potential of a validated cellular model of primary PCa was paralleled by enhanced FDG uptake and a marked decrease in the retention of a PSMA-targeted tracer. The preferential PSMA localization in the cytosol at least partially explained this finding.

Among the four identified glucose metabolism-related genes (PARP2, SLC2A4, CTH, ALDOB), at least two may directly corroborate the acknowledged predictive value of FDG PET imaging in PCa: ALDOB and SLC2A4. The former encodes for the glycolytic enzyme aldolase B, which has already been associated with poor clinical outcomes in several solid tumours [37] and represents a pivotal determinant of FDG uptake [38]. Similarly, the glucose transporter type 4 (GLUT4) encoded by the latter contributes to glucose uptake [39–41], predicts biochemical recurrence in the hormone-sensitive phase of the disease [42] and is increased in aggressive PCa phenotypes [41, 42]. On the other hand, no proven relationships with FDG uptake are currently reported in the literature for the two remaining genes identified by our bioinformatics analysis: PARP2 and CTH. However, PARP2 contributes to tumour aggressiveness by regulating several cellular functions, including the glycolytic rate [43]. Similarly, the enzyme cystathionine γ‐lyase, encoded by CTH, couples the capability to promote PCa spread with the activation of the Warburg effect via a mitochondrial impairment [44]. Although gene's expression may not exactly reflect protein translation levels and is only indirectly related to glucose consumption [45], the combined activation of these four genes agrees with the reported prognostic value of FDG uptake in PCa.

The present data suggest a more complex relationship between FOLH1 expression and PSMA-targeting tracers’ uptake. Since these two variables are strictly correlated in preclinical models [46], FOLH1 overexpression has been intuitively claimed to explain the predictive value of PSMA tracers’ uptake in PCa patients. However, in the present study, we found that FOLH1 expression does not predict PFS per se. The interplay between FOLH1 expression and PSMA-targeting tracers uptake depends upon protein localization since only plasma membrane PSMA is accessible for binding with the radioligand. The divergent tracer uptake in primary PCa cellular models with different biological aggressiveness agreed with the PSMA plasma membrane docking rather than the overall protein content (reflecting FOLH1 expression). This concept is corroborated by the specificity of the radioactive signal documented by the strict adherence of tracer uptake kinetics with the ligand-receptor model. Thus, in clinical studies assessing the prognostic value of PSMA PET imaging in early-stage PCa [10–13, 15, 47], the most common metrics adopted to quantify the PSMA-targeting tracer accumulation at PET imaging (i.e., SUV) may be influenced by several variables beyond FOLH1 expression, including PSMA-expressing cellular density or tumour burden. Based on our results, it can be hypothesized that the prognostic power of PSMA PET in this clinical setting might predominantly reflect these tumour characteristics rather than identifying a specific feature of PCa biological aggressiveness.

Finally, in the present study, we observed a decrease in the plasma membrane docking of PSMA in cellular models of aggressive primary PCa. Previous studies indicated that the most prominent PSMA membrane staining characterizes poorly differentiated primary PCa and distant metastases [48]. An acknowledged exception to this notion is the PCa neuroendocrine differentiation, in which FOLH1 expression has been shown to inversely correlate with neuron-specific enolase and somatostatin-receptor 2 in the pre-clinical analysis performed by Bakht et al. [49]. The present study suggests that neuroendocrine differentiation may not be the unique aggressive low-PSMA expressing variant of PCa, and that the downregulation of plasma membrane PSMA docking may represent a post-transcriptional source of heterogeneity at PSMA PET imaging, coherently with previous data [50]. Given the potential drawback to the diagnostic accuracy of this technique, further studies focusing on the complex relationship between the plasma membrane and cytosolic PSMA localization and tumour aggressiveness are thus needed.

Several limitations of our study deserve an accurate discussion. First, our multivariable model included gene expression exclusively. Therefore, we did not verify the eventual additive value of the obtained genetic score concerning conventional prognosticators in primary PCa, including Gleason Score, clinical stage, and PSA levels. Further studies are needed to address this point. Second, the predictive value of gene expression was analyzed in primary PCa. Accordingly, the present data cannot be extended to patients with metastatic and, even more importantly, castration-resistant diseases. This topic is relevant due to the increasing combined use of PSMA/FDG PET imaging in the clinical setting, e.g., in identifying ideal candidates for PSMA-based radioligand therapy [51]. Further studies addressing the gene expression profile underlying the divergent prognostic value of PSMA and FDG imaging in this clinical setting are thus still needed. Third, differential PCa aggressiveness was studied in the same cell line. This choice was based on previous studies documenting that studying cell cultures exposed to a low or a high passage number recapitulates the progression of human primary PCa towards a more aggressive disease [30, 31]. Forth, FOLH1 gene expression was not tested in these cultures that, however, showed a similar PSMA abundance associated with the reported difference in protein localization. Finally, the potential interference of androgen deprivation or androgen-receptor signalling inhibitors was not tested. Accordingly, further studies are needed to verify whether and to what degree these agents might interfere with the uptake of either FDG or PSMA and their prognostic values.

## Conclusion

The present study showed that the overexpression of a functional network involving four glucose metabolism-related genes (PARP2, SLC2A4, CTH, ALDOB) identifies a higher risk of disease progression since the earliest phases of PCa. Coupled with the observed tracer kinetics in two cellular models of different disease aggressiveness, this finding agrees with the acknowledged predictive value of FDG PET imaging in PCa. By contrast, the prognostic value of PSMA PET imaging is independent of the expression of its encoding gene FOLH1. Instead, it is influenced by the protein docking to the cellular membrane, regulating its accessibility to tracer binding. Altogether, these findings confirm that FDG and PSMA PET may provide complementary and independent prognostic information in newly diagnosed PCa, posing the bases for the design of clinical trials combining these tools for the PCa initial risk stratification.

## Supplementary Information

**Additional file 1: Table S1.** Glucose metabolism-related genes and FOLH1 interactors. **Table S2.** PARP2, SLC2A4, CTH and ALDO8 interactors downloaded from PINA

**Additional file 2.** Supplementary Methods and Figure S1 legend

**Additional file 3. Figure S1.**Functional network of PARP2, SLC2A4, CTH, ALDOB, and FOLH1 genes in PCa. Panel A: Functional network of the four genes, FOLH1 and their interactors reported in Supplementary Table 1 and Supplementary Table 2. The edge width is proportional to the correlation coefficient. The top 10 central genes of the network and their centrality score are listed in the inset table. Panel B: Graphical overview of ClueGO results of genes reported in Supplementary Table 2 and FOLH1 interactors (Supplementary Table 1). The pie diagram in the inset shows the percentage of the GO terms associated with the groups.

## Data Availability

The datasets analysed during the current study are available in the Protein Interaction Network Analysis (PINA) platform (https://omics.bjcancer.org/pina), and in the cBioPortal (https://www.cbioportal.org/) databases. The experimental data are available from the corresponding author upon reasonable request.

## Abbreviations

FDG: Fluorodeoxyglucose
GLUT4: Glucose transporter type 4
GO: Gene ontology
KEGG: Kyoto encyclopedia of genes and genomes
ML: Machine learning
PET/CT: Positron emission tomography/computed tomography
PCa: Prostate cancer
PFS: Progression-free survival
PINA: Protein interaction network analysis
PSMA: Prostate-specific membrane antigen
TCGA-PRAD: The cancer genome atlas prostate adenocarcinoma
