# Supplementary material for: Gene’s expression underpinning the divergent predictive value of [18F]F-fluorodeoxyglucose and prostate-specific membrane antigen positron emission tomography in primary prostate cancer: a bioinformatic and experimental study
Source: J Transl Med. 2023 Jan 4;21:3. doi: 10.1186/s12967-022-03846-1 (PMC9811737; doi:10.1186/s12967-022-03846-1)
Supplement: Supplementary file 2 — Additional file 2. Supplementary Methods and Figure S1 legend [file 12967_2022_3846_MOESM2_ESM.docx]

**Additional file Methods**

*Gene’s expression profile assessment*

Cancer Genome Atlas (TCGA) collected both tumour and non-tumour biospecimens from more than 10,000 human samples with informed consent under the authorisation of local Institutional Review Boards (https://cancergenome.nih.gov/abouttcga/policies/informedconsent). These steps ensured that patients were exposed to no unnecessary risks and that the research was legal, ethical, and well-designed. Among the TCGA available cohorts, we focused on the cancer genome atlas prostate adenocarcinoma (TCGA-PRAD) dataset. This allowed us to extract the raw signal for expression of the 122 preliminarily defined genes for each case following the TCGA pipeline from RNA Sequence data that uses MapSplice [1] to do the alignment and RSEM to perform the quantification [2].

For each individual tested gene, the mRNA level was expressed as a z-score, namely as the number of standard deviations away from the mean of expression in all sample’s diploids for the tested gene representing the reference population.

$$z-score= \frac{mRNA expression in tumor sample - mean mRNA expression in reference population}{standard deviation of mRNA expression in reference population}$$

**Additional file References**

[1] Wang K, Singh D, Zeng Z, Coleman SJ, Huang Y, Savich GL, et al. MapSplice: accurate mapping of RNA-seq reads for splice junction discovery. Nucleic Acids Res. 2010;38:e178.

[2] Li B, Dewey CN. RSEM: accurate transcript quantification from RNA-Seq data with or without a reference genome. BMC Bioinformatics. 2011;12:323.

**Additional file Figure Legend.**

**Additional file Figure S1: Functional network of PARP2, SLC2A4, CTH, ALDOB, and FOLH1 genes in PCa.** Panel A: Functional network of the four genes, FOLH1 and their interactors reported in Supplementary Table 1 and Supplementary Table 2. The edge width is proportional to the correlation coefficient. The top 10 central genes of the network and their centrality score are listed in the inset table. Panel B: Graphical overview of ClueGO results of genes reported in Supplementary Table 2 and FOLH1 interactors (Supplementary Table 1). The pie diagram in the inset shows the percentage of the GO terms associated with the groups.
